# An autocrine purinergic signaling controls astrocyte-induced neuronal excitation

**DOI:** 10.1038/s41598-017-11793-x

**Published:** 2017-09-12

**Authors:** Weida Shen, Ljiljana Nikolic, Claire Meunier, Frank Pfrieger, Etienne Audinat

**Affiliations:** 1 Inserm U1128, Paris Descartes University, 75006 Paris, France; 2 Institute of Cellular and Integrative Neurosciences, CNRS UPR 3212, University of Strasbourg, 67084 Strasbourg, France

## Abstract

Astrocyte-derived gliotransmitters glutamate and ATP modulate neuronal activity. It remains unclear, however, how astrocytes control the release and coordinate the actions of these gliotransmitters. Using transgenic expression of the light-sensitive channelrhodopsin 2 (ChR2) in astrocytes, we observed that photostimulation reliably increases action potential firing of hippocampal pyramidal neurons. This excitation relies primarily on a calcium-dependent glutamate release by astrocytes that activates neuronal extra-synaptic NMDA receptors. Remarkably, our results show that ChR2-induced Ca^2+^ increase and subsequent glutamate release are amplified by ATP/ADP-mediated autocrine activation of P2Y1 receptors on astrocytes. Thus, neuronal excitation is promoted by a synergistic action of glutamatergic and autocrine purinergic signaling in astrocytes. This new mechanism may be particularly relevant for pathological conditions in which ATP extracellular concentration is increased and acts as a major danger signal.

## Introduction

Astrocytes are the most abundant type of glial cells of the central nervous system and their position between blood vessels and synapses allows them to fulfill metabolic and homeostatic functions that are essential for the proper functioning of neuronal networks^1^. Evidence accumulated over the last two decades indicates that they also directly contribute to neuronal information processing through reciprocal interactions operating on relatively rapid time scales (ms to sec). These interactions rely first on the morphology of astrocyte processes that extend thin protrusions in the close vicinity of synaptic clefts and very often enwrap synapses^2–5^. The presence of specific transporters and receptors for neurotransmitters on these thin processes allows astrocytes to detect synaptic activity^1, 6–8^. In turn, astrocytes regulate the efficacy of synaptic transmission and neuronal excitability through the release of gliotransmitters^1, 9–11^. However, despite the growing number of publications in the field, the mechanisms, conditions and physiologic relevance of gliotransmitter release remain highly controversial. There is a general agreement that intracellular Ca^2+^ is a key signaling pathway through which astrocytes integrate the activation state of diverse membrane receptors. On the other hand, it is still controversial whether and how intracellular Ca^2+^ contributes to gliotransmitter release^12–16^.

A major challenge regarding astrocyte functions is to understand how these cells control and coordinate the release of the different gliotransmitters they produce. In the case of glutamate and ATP, two of the most widely studied gliotransmitters, both can regulate synaptic transmission and neuronal excitability^9^. However, it is unclear whether they are co-released by astrocytes, target the same cells or the same cellular compartments and have synergistic actions to promote excitation. Coupling between purinergic receptors P2Y1 (P2Y1Rs) and glutamate release in astrocytes has been described^17, 18^ but mechanisms of this coupling as well as the combined effect of astrocytic purinergic and glutamatergic signaling on neuronal activity remains poorly characterized. Finally, adenosine resulting from the degradation of astrocyte-derived ATP very often inhibits neurons and synaptic release^19^, making it difficult to predict what will be the net effect of glutamate and ATP co-release by astrocytes on neuronal network activity.

One important limitation in the field has been the difficulty to activate or inhibit astrocytes in a reliable and specific manner due to overlapping repertoire of membrane receptors with neurons. The development of new genetic models and pharmaco- and opto-genetic approaches enables new strategies to overcome this limitation^20^. In particular, optogenetic manipulations of astrocytes have confirmed their ability to release ATP and glutamate^21–29^. However, the questions related to the co-release of these gliotransmitters (see above) have not been addressed with these new approaches. There is therefore a need for a more refined dissection of the mechanisms governing gliotransmission in order to understand better the complex relationship between purinergic and glutamatergic signaling in astrocytes^30^.

Here, we have used a transgenic approach to express ChR2 in hippocampal astrocytes in a highly specific, widespread and reliable manner. In this model, blue light stimulation reliably triggered time-locked Ca^2+^ elevations in astrocytes and subsequent glutamate release that targets neuronal receptors. Remarkably, this astrocyte glutamate release is tightly controlled by an autocrine mechanism involving ATP release and P2Y1R activation. This synergistic action of purinergic and glutamatergic signaling in astrocyte promote neuronal excitation.

## Results

### ChR2 photoactivated astrocytes modulate neuronal activity

**Figure 1:**
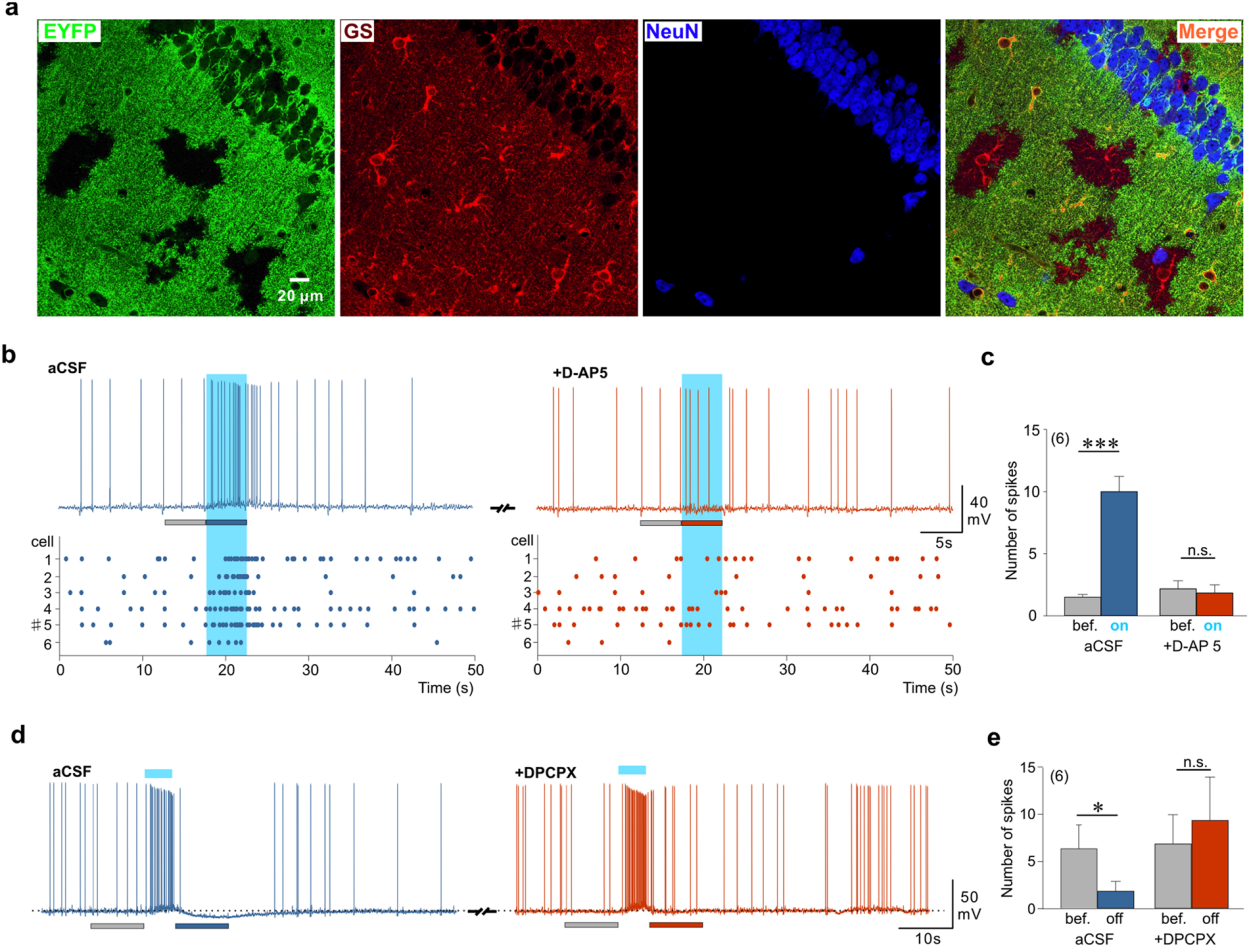
ChR2 photoactivation of astrocytes increases neuronal activity. (**a**) Immunohystochemical localization of ChR2-EYFP, glutamine synthetase (GS) and NeuN in the CA1 region of the hippocampus of C*x30-CreERT2*:*ChR2-EYFP* mice. ChR2-EYFP expression is selectively confined to GS-expressing astrocytes and not to NeuN immunoreactive neurons. (**b**) Current clamp traces and corresponding raster plots illustrating the responses of CA1 neurons to light activation of astrocytes (light blue marks in all figures) before and after application of 50 µM D-AP5. The traces shown correspond to cell number 5 in the raster plots. A constant depolarizing current was injected throughout recordings to bring membrane potential near action potential threshold. (**c**) Number of action potentials during 5 s time windows before and during photostimulation of astrocytes in control conditions and in the presence of D-AP5 (control: t(5) = 7.603, P = 0.0006; D-AP5: t(5) = 0.674, P = 0.53; 6 cells, 3 animals, paired t-test). (**d**) Example of a CA1 pyramidal neuron responding to light stimulation of astrocytes by an excitation-inhibition sequence and block of the inhibition by the adenosine A1 receptor antagonist DPCPX (300 nM). (**e**) Number of action potentials during 10 s time windows before and after photostimulation of astrocytes in control conditions and in the presence of DPCPX (control: t(5) = 2.957, P = 0.032; DPCPX: t(5) = 1.431, P = 0.212, 6 cells, 4 animals, paired t-test). For all figures n.s. P > 0.05, *P < 0.05, **P < 0.01, ***P < 0.001.

In order to study the consequences of astrocyte stimulation on neuronal activity we used a transgenic approach aiming at expressing light-sensitive ChR2 specifically in astrocytes. We crossed *floxed-ChR2-EYFP* and *Cx30-CreERT2* mice (Supplementary Fig. 1a) that target hippocampal astrocytes^31^. We observed that a majority of astrocytes identified by the specific markers glutamine synthase (GS; Fig. 1a) or glial fibrillary acidic protein (GFAP; Supplementary Fig. 1b) were also positive for EYFP and therefore likely expressed ChR2. On average, we found that 69.44 ± 7.43% (119 cells from 3 animals) of astrocytes immunopositive for GS in the CA1 region were also immunopositive for EYFP. Conversely, we did not observe co-localization between NeuN labeled neurons and EYFP (Fig. 1a). In acute hippocampal slices, cells expressing ChR2-EYFP exhibited passive membrane properties typical of astrocytes (Supplementary Fig. 1c) and were reliably activated by blue light: at a holding potential of −80 mV, blue light stimulations induced inward currents with short latencies (in the ms range) and rise times (~5 ms) that were proportional to the intensity of light and lasted for the entire duration of light stimulation (Supplementary Fig. 1d). In agreement with the above described immunohistochemical data, we never recorded rapid ChR2-mediated currents in hippocampal CA1 neurons (see below). Thus, light induces ChR2 activation specifically in astrocytes.

We then took advantage of this widespread and specific astrocytic expression of ChR2 to assess the consequences of astrocyte activation on neuronal activity. In the absence of pharmacological agents, when maintaining CA1 pyramidal cells near their threshold for action potentials (around −50 mV), photoactivation of astrocytes evoked a small depolarization accompanied by an increase in action potential firing (Fig. 1b,c). Because excitatory gliotransmission often relies on the release of glutamate acting at NMDA receptors (NMDARs) or metabotropic receptors (mGluRs)^9^, we first tested the effect of antagonists of these receptors. We observed that the light-induced increase in action potential firing was prevented by the NMDAR antagonist D-AP5 (Fig. 1b,c) but not by co-applying mGluR antagonists MPEP (50 µM), CPPG (100 µM) and LY 367385 (100 µM: increase in spike number in aCSF 334 ± 67%, t(7) = 3.491, P = 0.01, 8 cells, 4 animals) and with mGluR antagonists 439 ± 82%, t(7) = 4.114, P = 0.004, 8 cells, 4 animals). We also excluded a contribution of a down-regulation of postsynaptic GABA_A_ receptors^32^ and of a modulation of synaptic transmission via the activation of pre- or post-synaptic P2X receptors^33–35^, that contribute to the astrocytic control of neuronal excitability (Supplementary Fig. 2). These observations suggest that astrocyte photostimulation enhances CA1 neuron excitability via the activation of NMDARs.

In 10 out of 26 neurons recorded in control conditions (*i*.*e*. in absence of any receptor antagonists), we observed that the initial depolarization induced by astrocyte photoactivation was followed by a hyperpolarization that usually peaked after cessation of the light pulse and inhibited action potential firing of CA1 neurons (Fig. 1d). The antagonist of adenosine A1 receptors, DPCPX, abolished this delayed inhibition of firing (Fig. 1e) without affecting the increase in firing occurring during the light pulse (number of spikes 15.66 ± 4.68 in control conditions versus 15.16 ± 4.26 in DPCPX, t(5) = 0.183, *P* = 0.862, 6 cells, 4 animals, paired t-test). In agreement with previous observations^29^, this suggests that astrocyte photoactivation leads to the release of ATP that is degraded into adenosine and inhibits CA1 pyramidal cells. Taken together our data indicate that astrocyte photoactivation induces the release of glutamate that excites CA1 pyramidal neurons and that a secondary production of adenosine is responsible for their delayed inhibition.

### Gliotransmission triggered by astrocyte photostimulation excites CA1 neurons by activating postsynaptic NMDA receptors

**Figure 2:**
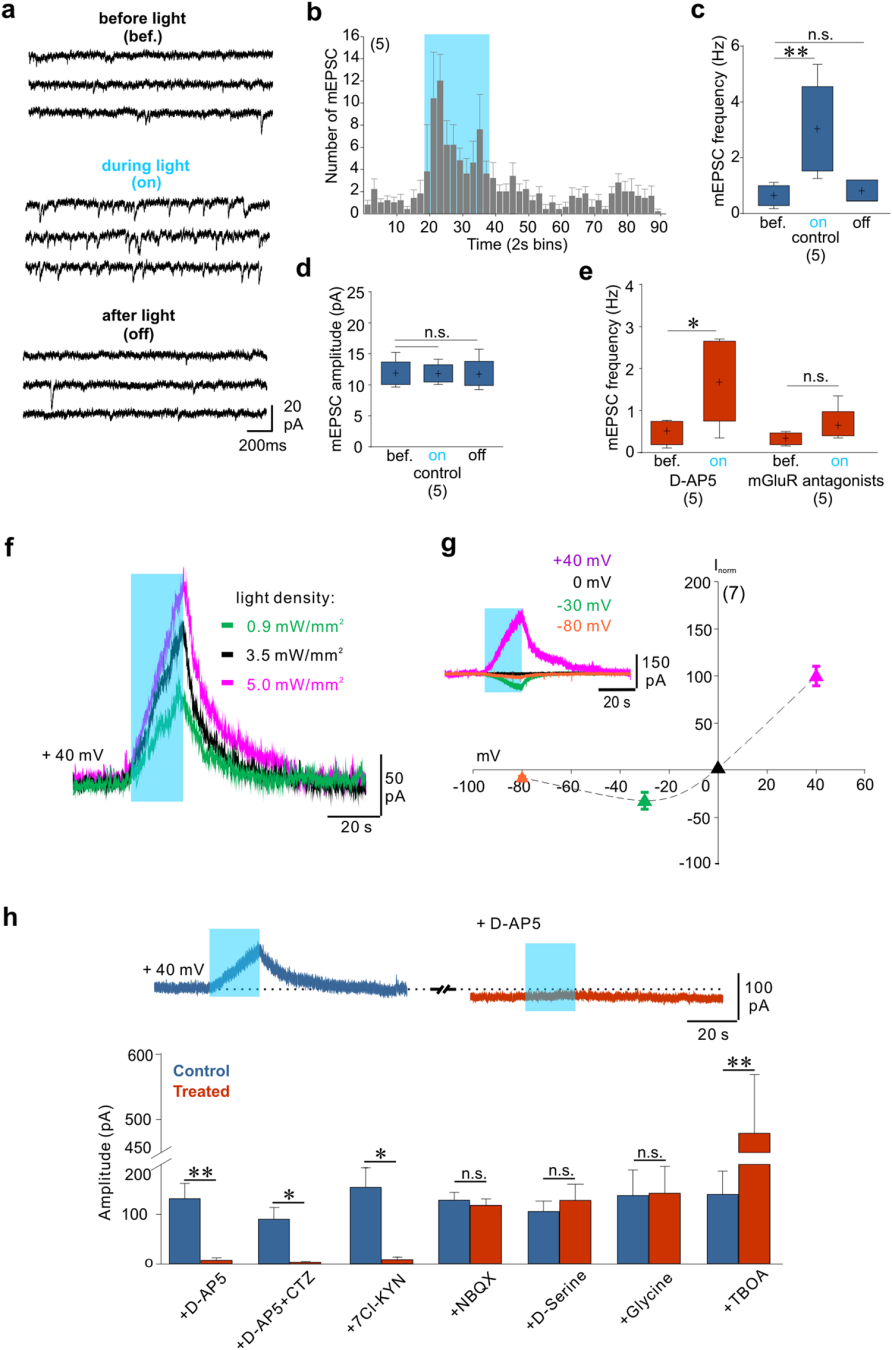
Pre- and postsynaptic effects induced in CA1 neurons by astrocyte glutamate release. (**a**) Current traces illustrating the increase in mEPSC frequency evoked by astrocyte photoactivation. (**b**) Time course of light-induced changes in mEPSC number (bin width 2 s). (**c**,**d**) Effect of astrocyte photostimulation on the frequency and amplitude of mEPSCs (One-way RM ANOVA; Frequency: F(2,8) = 14.382, P = 0.002, Bonferroni post-hoc: P = 0.003 (bef. versus on), P = 1 (bef. versus off); Amplitude: F(2,8) = 0.019, P = 0.981; 5 cells, 2 animals). (**e**) Differential effect of D-AP5 and mGluR antagonists on the mEPSC frequency during light stimulation. (50 µM D-AP5: t(4) = 3.267, P = 0.031; mGluR antagonists: 100 µM LY367385, 100 µM CPPG, 50 µM MPEP: t(4) = 1.774, P = 0.151, 5 cells, 2 animals), paired t-tests. (**f**) Astrocyte photostimulation evokes tonic current in CA1 neurons proportional to the density of light. (**g**) I–V curve of the evoked tonic current (normalized to the value at +40 mV, 7 cells, 3 animals). Inset: light-evoked current at different holding potentials. (**h**) Representative traces showing the inhibition of the tonic current by D-AP5. Pharmacological characterization of the tonic current induced by astrocyte photostimulation: D-AP5 (50 µM: t(10) = 4.433, P = 0.001, 11 cells, 5 animals), Cyclotiazide (25 µM CTZ: t(4) = 3.803, P = 0.019, 5 cells, 3 animals), 7-Chlorokynurenic acid (50 µM 7Cl-KYN: t(4) = 4.201, P = 0.014, 5 cells, 3 animals), NBQX (10 µM: t(8) = 0.773, P = 0.462, 9 cells, 3 animals), D-Serine (10 µM: t(9) = 1.635, P = 0.137, 10 cells, 5 animals), Glycine (100 µM: t(4) = 1.091, P = 0.337, 5 cells, 3 animals), and TBOA (100 µM: t(4) = 4.937, P = 0.008, 5 cells, 3 animals), paired t-test.

Next we sought to understand whether the modulation of neuronal activity involving NMDAR activation was mediated by pre- or postsynaptic mechanisms. We first tested the effect of astrocyte photostimulation on the frequency of miniature excitatory postsynaptic currents (mEPSCs) in CA1 neurons. In the presence of tetrodoxin (TTX, 0.5 µM) and GABAzine (GBZ, 10 µM) astrocyte stimulation induced an increase in mEPSC frequency without changing mEPSC amplitude (Fig. 2a–d), suggesting a presynaptic effect. Consistent with previous results^36^, this effect was mediated by mGluRs but not by NMDARs (Fig. 2e). Our data therefore show that astrocyte photostimulation increase presynaptic glutamate release through the activation of mGluRs but this mechanism does not contribute significantly to the increase in CA1 neurons firing, which is mediated by NMDAR activation. We then explored whether astrocyte modulation of neuronal activity could involve postsynaptic NMDARs. Indeed, we and others previously reported that manipulations known to favor the release of glutamate from astrocytes induce a tonic current in CA1 hippocampal neurons via the activation of extrasynaptic NMDARs^37–39^. We recorded CA1 pyramidal cells at a holding potential of +40 mV in the presence of 0.5 µM TTX and 10 µM GBZ. Shining blue light induced a slowly developing outward current, the amplitude of which increased as long as the light stimulation was maintained and was proportional to the light intensity (Fig. 2f). This tonic current had the typical current/voltage relationship of NMDARs, with a negative slope at potentials more hyperpolarized than −30 mV (Fig. 2g), indicating that NMDARs responsible for this tonic current were expressed by the recorded neurons. The tonic current was also inhibited by bath application of the NMDAR antagonist D-AP5 (Fig. 2h). Note also that D-AP5 decreased the steady state holding current (Fig. 2h). Pharmacological characterization of the light-induced tonic current further pointed toward the activation of only NMDARs by astrocyte release of glutamate. The light-induced tonic current was not inhibited by the AMPA/kainate receptor antagonist NBQX (Fig. 2h) and the inhibitor of AMPA receptor desensitization cyclotiazide (CTZ) did not reveal an additional component (Fig. 2h). In addition, the light-induced current was inhibited by the antagonist of the glycine/D-serine site of NMDARs 7-Chlorokynurenic acid (7Cl-KYN) (Fig. 2h). However, inconsistent with a release of D-serine or glycine from astrocytes, the light-induced tonic current was not affected by bath application of glycine or D-serine (Fig. 2h), which in our conditions did not increase either the steady-state holding current (see also ref. 37). Conversely, the light-induced current was greatly increased upon application of the glutamate uptake inhibitor DL-TBOA (Fig. 2h), which also increased the steady-state holding current (not shown but see ref. 37). These results indicate that activation of astrocytes induces glutamate release that regulates CA1 neuron activity through tonic activation of neuronal NMDARs. The tonic NMDAR-mediated current induced by ChR2 stimulation of astrocytes was not inhibited by incubating slices in bafilomycin A1 that totally inhibited neuronal vesicular release (Supplementary Fig. 3a,b; see also ref. 37). In contrast, applying 100 µM of 5-nitro-2-(3-phenylpropyl amino) benzoic acid (NPPB), an antagonist of Ca^2+^-dependent chloride channels Bestrophin-1 (Best1)^40^, inhibited the NMDAR-mediated tonic current (Supplementary Fig. 3c), suggesting that the glutamate release induced by ChR2 stimulation in astrocyte was mediated through a non-vesicular, Best1-dependent mechanism.

Next, we tested whether glutamate release from light-activated astrocytes target extra-synaptic or synaptic NMDARs^37, 41^. Extra-synaptic NMDARs of CA1 neurons contain GluN2B subunits that are sensitive to the modulator Ro 25–6981^41–43^. Bath application of Ro 25–6981 enhanced light-induced currents (Supplementary Fig. 4a) but had no effect on the NMDAR component of EPSCs elicited by the electrical stimulation of Schaffer collaterals (SC, Supplementary Fig. 4b). NMDARs at these SC-CA1 synapses contain GluN2A subunits that can be inhibited by nanomolar concentrations of extracellular zinc^44–46^. Bath application of 300 nM free zinc (see Methods) readily inhibited the NMDAR-mediated component of evoked SC-CA1 EPSCs (Supplementary Fig. 4d) but had no effect on the amplitude of the light-induced current (Supplementary Fig. 4c). The subunit composition of NMDARs mediating the light-induced current therefore differs from that of NMDARs at SC-CA1 synapses. Moreover, the observation that Ro 25–6981 potentiated rather than inhibited the light-induced current indicates that the concentration of glutamate reaching extra-synaptic receptors is low, probably in the range of 50 to 500 nM^37, 39, 42, 47^.

### Autocrine ATP signaling shapes Ca^2+^ signals in astrocytes

**Figure 3:**
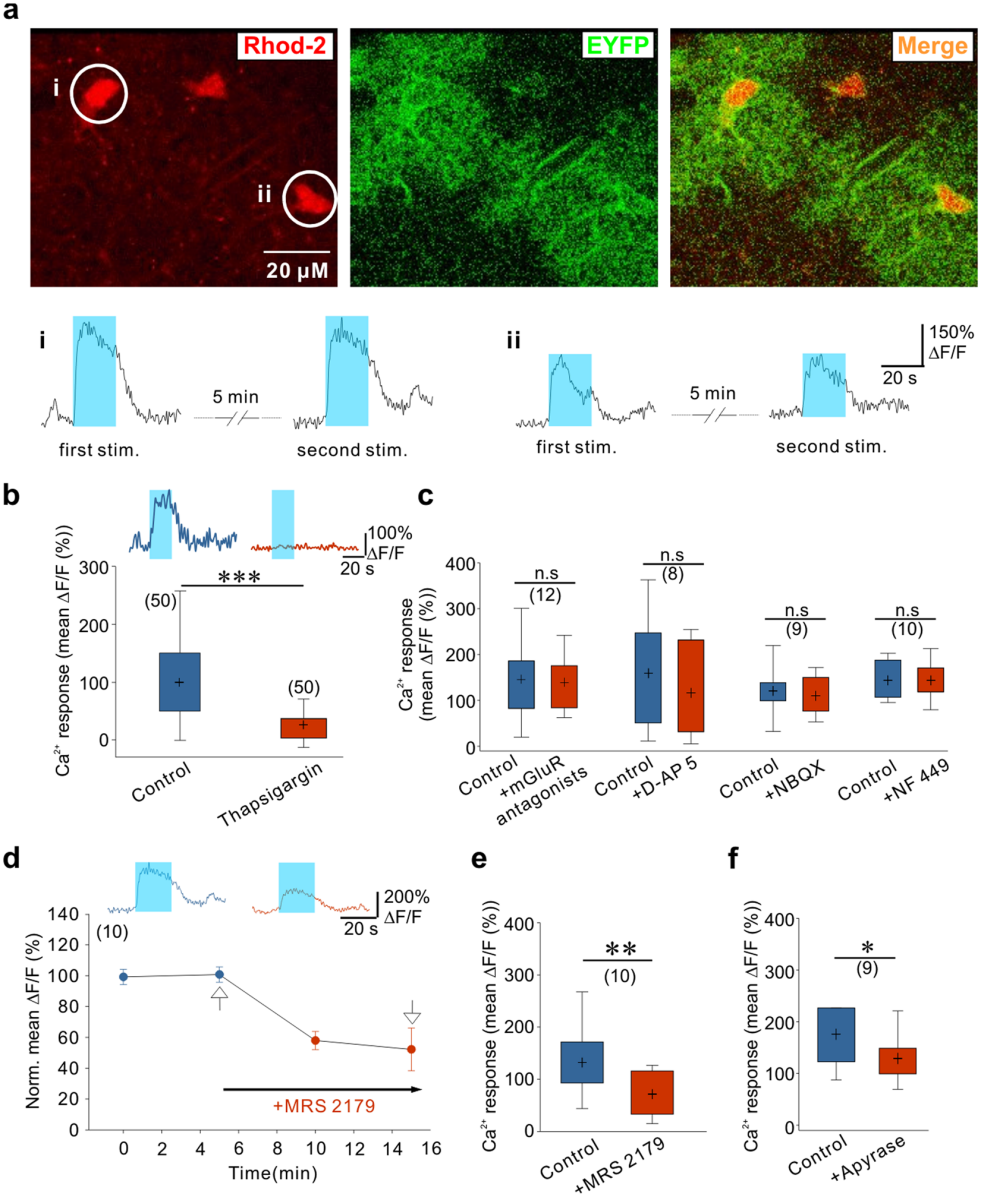
ATP-dependent Ca^2+^ responses induced by astrocyte photostimulation. (**a**) Two-photon images showing Rhod-2 (red) loaded CA1 astrocytes expressing EYFP (green) in a hippocampal slice. Lower panel, changes in Rhod-2 emission (ΔF/F) intensity monitored in the soma of 2 astrocytes (marked by i and ii) in response to 2 successive photostimulations. (**b**) Slices pre-treatment with thapsigargin (1 µM) decreased the light-induced Ca^2+^ elevations in EYFP-positive astrocytes as compared to control slices (t(98) = 7.772, P < 0.0001, 100 cells, 5 animals, unpaired t-test). Upper traces represent calcium signals evoked by photostimulation in control (left) and thapsigargin (right) treated slices. (**c**) Effects of glutamate receptor antagonists on light-induced Ca^2+^ responses in EYFP-positive astrocytes: mGluR antagonists (100 µM LY367385, 100 µM CPPG, 50 µM MPEP: t(11) = 0.457, P = 0.657, 12 cells, 4 animals), DAP-5 (50 µM: t(7) = 0.907, P = 0.395, 8 cells, 3 animals), NBQX (10 µM: t(8) = 0.777, P = 0.46, 9 cells, 3 animals), NF 449 (10 µM: t(9) = −0.005, P = 0.996, 10 cells, 2 animals), paired t-test. (**d**) Time-course of MRS 2179 evoked change in light-induced Ca^2+^ signals in EYFP-positive astrocytes. Examples of Ca^2+^ responses at time points indicated by the arrows are shown above the graph. (**e**) Effect of 10 µM MRS 2179 on light-evoked Ca^2+^ responses (t(9) = 3.562, P = 0.006, 10 cells, 6 animals, paired t-test). (**f**) Effect of the ATP/ADP degrading enzyme apyrase on the amplitude of light-evoked Ca^2+^ responses in astrocytes (25 U/ml: t(8) = 3.029, P = 0.016, 9 cells, 2 animals, paired t-test).

To analyze the mechanisms through which photostimulated astrocytes release glutamate, we first investigated the role of astrocytic calcium signals by two-photon imaging of hippocampal slices bulk-loaded with the membrane-permeant form of the red calcium fluorescent indicator Rhod-2 (see Methods and Fig. 3a). Only a minority of EYFP positive astrocytes were co-labelled with Rhod-2 and in the presence of TTX and GBZ, to decrease the influence of neuronal activity, most of them exhibited spontaneous somatic Ca^2+^ transients (see Fig. 3a,b). Blue light stimulations induced somatic Ca^2+^ elevations in EYFP-expressing astrocytes with mean amplitude of 226.06 ± 15.06 deltaF/F that peaked 5.89 ± 0.34 seconds after the onset of the stimulation (43 cells, 14 animals). These Ca^2+^ responses were maintained during the entire duration of the light stimulation and decreased with a time constant of 17.91 ± 1.29 seconds after the end of the stimulation (Fig. 3a, lower panels). Considering the amplitude and the time course of these Ca^2+^ transients, and the relatively low Ca^2+^ permeability of ChR2^48–50^, we hypothesized that mechanisms downstream of ChR2 activation could recruit Ca^2+^ release from internal stores. We therefore pre-incubated slices with thapsigargin to deplete intracellular Ca^2+^ stores. Consistent with our hypothesis, we observed that the amplitude of the Ca^2+^ transients triggered by ChR2 stimulation in thapsigargin-treated slices was greatly reduced as compared with interleaved control slices from the same animals (Fig. 3b). Intracellular Ca^2+^ stores in astrocytes can be mobilized through the activation of several membrane receptors. Since the results described above indicated that photostimulation of astrocytes induces the release of glutamate and ATP, we thus sought to determine whether activation of astrocyte receptors contributes to these ChR2-induced Ca^2+^ responses. We first tested the involvement of glutamate receptors. However, bathing the slices with a cocktail of mGluR antagonists or with an antagonist of NMDARs or of AMPA/kainate receptors did not decrease the amplitude of the ChR2-induced Ca^2+^ transients in astrocytes (Fig. 3c). Next we examined the involvement of purinergic receptors. P2Y1Rs are highly expressed by astrocytes and are potent stimulant of Ca^2+^ release from intracellular stores in these cells^51^. As shown in Fig. 3d, MRS 2179, an antagonist of P2Y1Rs, strongly inhibited the amplitude of astrocyte Ca^2+^ signals induced by ChR2 activation. Although selective for P2Y1Rs, MRS 2179 at a concentration of 10 µM can also inhibit P2X_1_ and P2X_3_ receptors, which likewise participate in astrocyte Ca^2+^ signaling^51, 52^. Therefore, we further examined the contribution of these P2XR subtypes to the ChR2-induced Ca^2+^ signals in astrocytes. Application of NF 449 at a concentration (10 µM) that blocks P2X_1_ and P2X_3_ receptors, however, had no effect on astrocyte Ca^2+^ responses evoked by light stimulation (Fig. 3c). Altogether, these data indicate that Ca^2+^ elevation evoked during photoactivation of astrocytes is largely due to an autocrine ATP signaling involving selectively P2Y1Rs and mobilization of intracellular Ca^2+^ stores. The involvement of purinergic signaling was further supported by the observation that the ATP/ADP degrading enzyme apyrase also decreased the amplitude of Ca^2+^ transients induced by ChR2 activation in astrocytes (Fig. 3f).

### Autocrine P2Y1R activation regulates astrocyte-induced neuronal excitation

**Figure 4:**
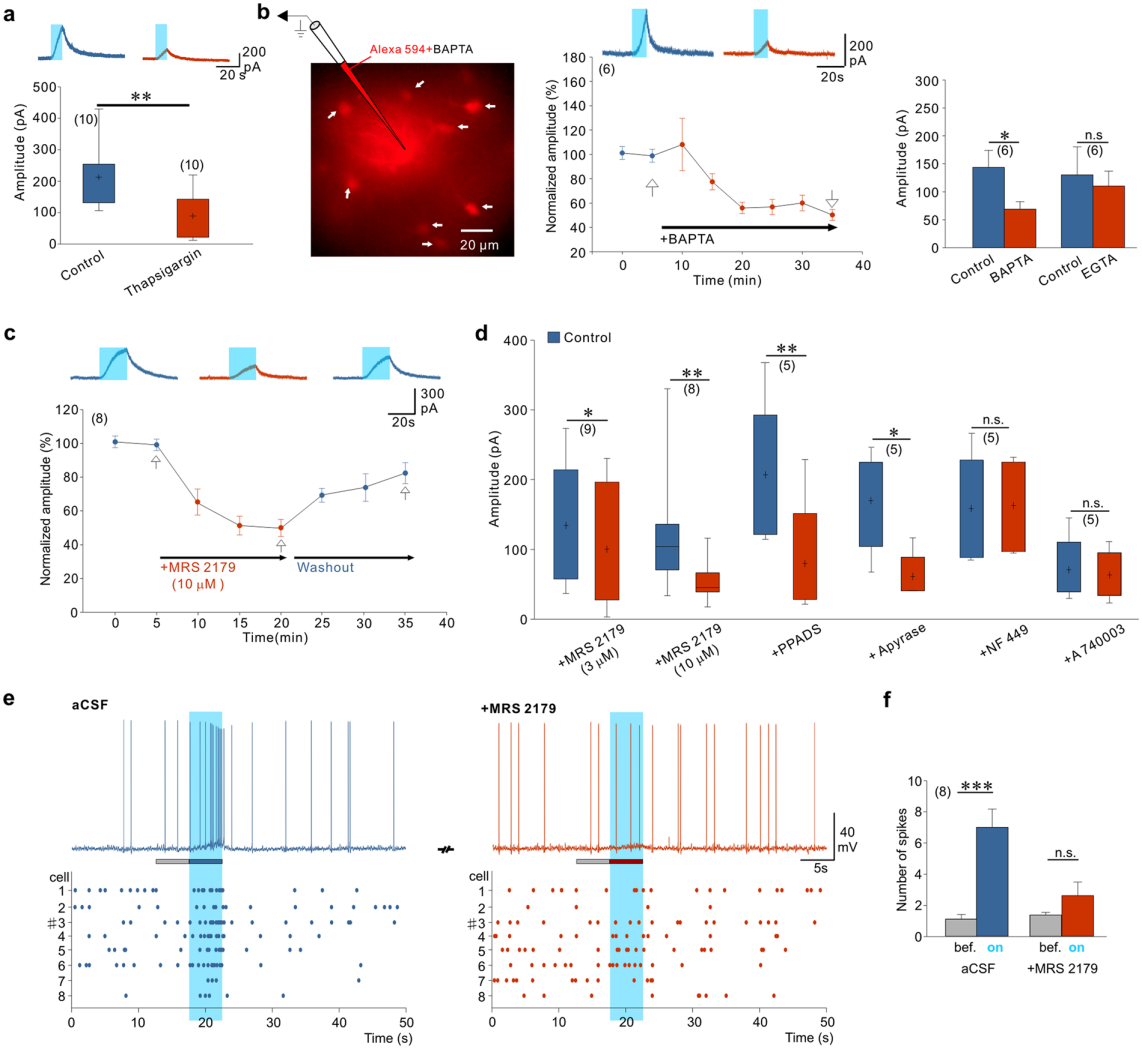
Autocrine activation of P2Y1R on astrocytes potentiates neuronal activity. (**a**) Emptying Ca^2+^ stores by thapsigargin (1 µM) decreased the light-induced tonic NMDAR-mediated current (t(18) = 3.126, P = 0.006, 20 cells, 4 animals, unpaired t-test). Upper traces represent the effect of thapsigargin on the tonic NMDA current evoked by astrocyte photostimulation. (**b**) Left, syncytium of astrocytes (arrows) in CA1 stratum radiatum labeled with Alexa 594 after 30 min of recording of a single astrocyte with a pipette containing BAPTA (40 mM) and Alexa 594 (25 µM). Middle, time course of BAPTA effect on normalized NMDAR-mediated tonic current. Right, 30 min of BAPTA dialysis significantly reduced the amplitude of tonic NMDAR-mediated current (t(5) = 3.330, P = 0.021, 6 cells, 4 animals) whereas 30 min of low EGTA dialysis had no effect (t(5) = 0.424, P = 0.689, 6 cells, 3 animals), paired t-test. (**c**) Time course of the effect of P2Y1R antagonist MRS 2179 on the amplitude of the light-induced tonic NMDAR-mediated current in CA1 neurons. Examples of light-evoked tonic currents at +40 mV at time points indicated by arrows are shown above the graph. (**d**) P2Y1R-mediated purinergic signaling controls tonic glutamate release from astrocytes. The amplitude of the light-induced NMDAR-mediated currents is significantly reduced by MRS 2179 (3 µM: t(8) = 2.543, P = 0.035, 9 cells, 4 animals, paired t-test; 10 µM: z = 2.521, P = 0.008, 8 cells, 3 animals, Wilcoxon test﻿), PPADS (100 µM: t(4) = 4.682, P = 0.009, 5 cells, 3 animals, paired t-test) and apyrase (25 U/ml: t(4) = 3.774, P = 0.020, 5 cells, 3 animals, paired t-test). Light-evoked NMDAR-mediated currents are not affected by P2XRs antagonists NF 449 (10 µM: t(4) = 0.391, P = 0.716, 5 cells, 3 animals, paired t-test) or P2X_7_R antagonist A 740003 (20 µM: t(4) = 1.114, P = 0.328, 5 cells, 3 animals, paired t-test). (**e**) Current clamp traces and corresponding raster plots illustrating the response of CA1 neurons to light activation of astrocytes before and after application of MRS 2179. The traces shown correspond to cell number 3 in the raster plots. (**f**) Effect of 10 µM MRS 2179 on the number of action potentials during 5 s time windows before and during astrocyte photostimulations (control: t(7) = 6.289, P = 0.0004; MRS 2179: t(7) = 1.357, P = 0.217, 8 cells, 3 animals, paired t-test).

We then investigated whether Ca^2+^ elevation in astrocytes is required to evoke tonic NMDAR-mediated current in CA1 neurons. By depleting Ca^2+^ stores with thapsigargin, we observed that the amplitude of the light-induced tonic NMDA current was smaller than in control slices (Fig. 4a). Yet, to prove directly that astrocyte Ca^2+^ was necessary for the release of glutamate, we monitored the NMDAR-mediated tonic current triggered by ChR2 stimulations while dialyzing, through a second patch-pipette, the astrocytic syncytium with the Ca^2+^ chelator BAPTA (inter-pipette distance 69.8 ± 16.9 µm, 6 paired recordings). Alexa 594 was included in the astrocyte pipette to verify the extent of the dialysis to neighboring astrocytes via gap junctions (Fig. 4b). After 15 min of dialysis, this dye coupling usually reached astrocytes at a distance of approximately 80 µm from the injected cell. The amplitude of the evoked tonic NMDAR-mediated current decreased with time along with the dialysis of BAPTA in astrocytes (Fig. 4b). In contrast, when BAPTA was replaced by a low concentration of EGTA, the tonic NMDA current was stable (Fig. 4b; inter-pipette distance 56.6 ± 9.18 µm, 6 paired recordings). These observations indicate that increase in cytoplasmic Ca^2+^ favors astrocyte glutamate release. ChR2 is highly permeable to Na^+^ and H^+^^49^. In additional experiments we excluded that ChR2-induced Ca^2+^ elevation and subsequent astrocyte glutamate release are mediated by the reversal operation of the Na^+^/Ca^2+^ exchanger following Na^+^ influx through ChR2 channels (Supplementary Fig. 5a). Intracellular acidification during ChR2 activation may have contributed to the light-induced release of glutamate, as previously shown in the cerebellum^53^. Using the same protocol to decrease the proton buffer capacity of astrocytes than the one used in this previous study^53^, however, did not increase in our hands the amplitude of the tonic NMDAR-mediated current induced by light (Supplementary Fig. 5b).

Since Ca^2+^ elevation in astrocytes is controlled by purinergic signaling (see above), we therefore tested whether glutamate release and subsequent activation of NMDARs were also regulated by ATP release and activation of purinergic receptors in astrocytes. Light-induced tonic NMDAR-mediated current in CA1 neurons was inhibited by apyrase (Fig. 4d) and by MRS 2179 applied at concentrations of 3 µM (32.15 ± 12.02% of block) and 10 µM (52.62 ± 4.43% of block) indicating that P2Y1R activation by extracellular purines positively regulates astrocyte glutamate release (Fig. 4c,d). Broad spectrum P2 antagonist PPADS also blocked the light-induced tonic NMDAR-mediated current (Fig. 4d). We excluded any direct effect of P2YR antagonists^54^ on NMDARs: tonic currents evoked by NMDA applications were not changed by addition of either PPADS or MRS 2179 (Supplementary Fig. 6). Furthermore, we excluded the involvement of P2X_1_, P2X_3_ as well as P2X_7_ receptor subtypes, as blocking these receptors by NF 449 and A740003, respectively, had no effect on the amplitude of light-evoked NMDAR-mediated tonic current (Fig. 4d).

If the tonic NMDAR-mediated current relies on ATP dependent Ca^2+^ signaling in astrocytes, then the light-induced increase in action potential firing should also be dependent on activation of P2Y1Rs. Consistent with this, we observed that the increased discharge of CA1 pyramidal neurons during photostimulation is also blocked by antagonizing P2Y1Rs with MRS 2179 (Fig. 4e,f).

## Discussion

**Figure 5:**
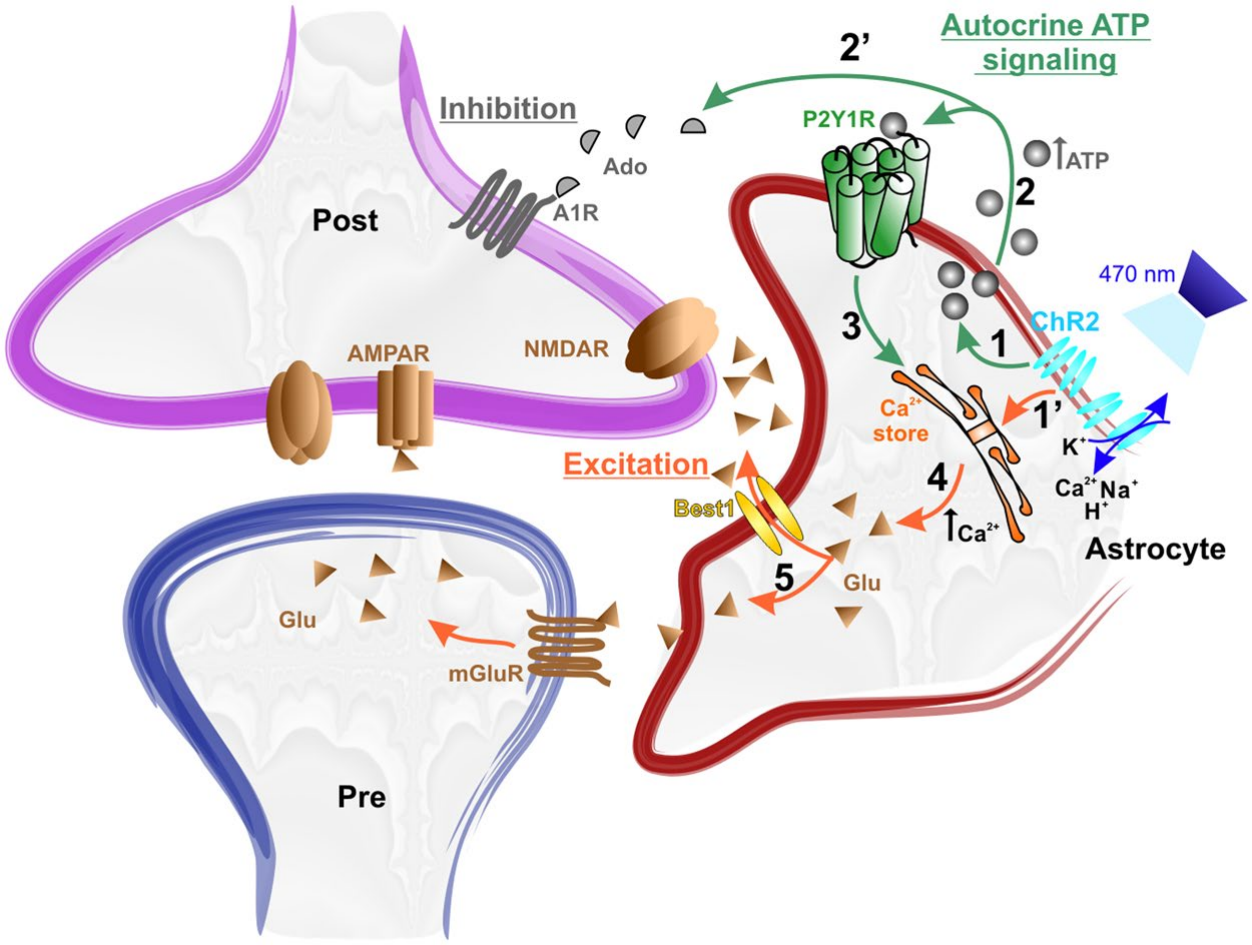
Autocrine ATP signaling pathway controls astrocyte glutamate release and neuronal activity. Schematic drawing summarizing the presented data: (1) photoactivation induces release of ATP from astrocytes; (2) released ATP activates P2Y1R via autocrine mechanism; (3) P2Y1R signaling pathway amplifies Ca^2+^ release from Ca^2+^ store; (4) increase in Ca^2+^ triggers release of glutamate (Glu) from astrocytes probably through bestrophin-1 channels (Best1); (5) Glu activates presynaptic mGluR and postsynaptic NMDAR, the latter being responsible for the increase in action potential firing of CA1 pyramidal neurons. (1′) ChR2 activation may directly mobilize Ca^2+^ stores and cause glutamate release; (2′) released ATP is also hydrolyzed in adenosine (Ado) that activates A1 receptors and induces inhibition of CA1 firing.

We have shown here that ChR2 activation in astrocytes is a reliable and useful tool to study gliotransmission. In the CA1 region of the hippocampus, photostimulation of astrocytes induces a release of glutamate that targets neuronal presynaptic mGluRs and postsynaptic NMDARs, the latter being responsible for a modulation of action potential firing of pyramidal neurons. Tonic activation of NMDARs in hippocampal neurons had been previously reported^55^ and indirect evidence pointed toward a glial origin of the ambient glutamate that activates these NMDARs^37–39^. Selective activation of astrocytes with optogenetics conclusively demonstrates that these glial cells contribute to the accumulation of ambient glutamate in the hippocampus, as is probably also the case in the cerebellum^23^. Remarkably, we observed that this glutamate release was largely dependent on astrocyte Ca^2+^ signaling and on an autocrine mechanism involving the release of ATP by astrocytes and the activation of astrocytic P2Y1Rs (summarized in Fig. 5). A paracrine mechanism, *i*.*e*. astrocyte ATP release activating P2Y1Rs of neighboring astrocytes, is most probably also involved but its identification will require further investigations.

Our data indicate that the glutamate release evoked by ChR2 stimulation in astrocytes is dependent on intracellular Ca^2+^. However, the peak of the Ca^2+^ responses induced in astrocytes by ChR2 stimulation is observed at an earlier time point than the peak of the tonic NMDAR-mediated current. This suggests that limiting mechanisms, downstream of Ca^2+^ responses, impose the kinetics of the NMDAR-mediated currents. Uptake of glutamate, for instance, is likely to control the kinetics of glutamate accumulation in the extracellular space and thus the kinetics of the NMDAR-mediated current. Mobilization of Ca^2+^ stores is an important step in the process and the P2Y1R-dependent autocrine signaling pathway boosts this mobilization. However, the initial step that leads from the ChR2 opening to the release of ATP is less clear. The Ca^2+^ permeability of ChR2 is low (P_Ca_/P_Na_ ~0.1)^49, 56^ and in cultured astrocytes ChR2 activation triggers Ca^2+^ signals that are less reliable and less efficient to induce gliotransmission than activation of other optogenetic actuators with a higher Ca^2+^ permeability^24^. It is still possible, however, that *in situ* the low Ca^2+^ permeability of ChR2 is sufficient to trigger ATP release and autocrine signaling or that a Ca^2+^-induced Ca^2+^ release initiates the release of ATP. An alternative hypothesis is that the flow of other ions through ChR2, namely Na^+^ and H^+^, triggers indirectly a subsequent Ca^2+^ increase or an initial Ca^2+^-independent release of ATP that would then activate the autocrine purinergic pathway. We excluded the contribution of a reverse operation of the Na^+^/Ca^2+^ exchanger and that of an intracellular acidification (Supplementary Fig. 5). Yet, an initial ATP release could occur through volume-regulated anion channels^57^ or through a transporter^58^, as a cell adaptation mechanism to the change in intracellular osmolality during light stimulation. It should be also stressed that part of the Ca^2+^ response and of the glutamate release induced by ChR2 stimulation is not blocked by inhibiting ATP signaling. This suggests that a direct pathway, linking ChR2 to Ca^2+^ stores and to glutamate release, independently of ATP signaling, operates in parallel.

In any case, ChR2 reliably triggered large somatic Ca^2+^ elevations resembling those occurring simultaneously in astrocyte populations in specific physiological conditions *in vivo*. This is the case of Ca^2+^ transients evoked by pH changes in astrocytes of the chemosensitive area of the ventral surface of the medulla oblongata^21^ and of Ca^2+^ transients evoked by norepinephrine in astrocytes of several brain regions during startle behaviors^59, 60^. Simultaneous ChR2-induced activation of groups of astrocytes may therefore represent a good model to study the consequences of this type of Ca^2+^ signals on astrocyte-to-neuron communication. Our observations predict that the ATP-P2Y1R autocrine loop is an important modulator of this form of Ca^2+^ signaling. The use of Rhod2-AM, which labels mostly cell bodies, has prevented us from analyzing Ca^2+^ signaling in the thin processes of astrocytes in response to ChR2 photostimulation. However, Ca^2+^ micro-domains in astrocyte processes are major determinants of astrocyte-dependent regulation of synaptic transmission and neuronal excitability^61, 62^ and further studies are therefore needed to evaluate their role in the form of gliotransmission that we describe here.

There is so far no clear picture emerging from the few studies that have used optogenetics to analyze how astrocytes modulate neuronal and synaptic networks. On the one hand, ChR2 stimulation of astrocytes in the cerebellum and the neocortex induces a release of glutamate^23, 26, 53^. This release evoked by optogenetic activation of cerebellar Bergmann glia leads to the activation of AMPA receptors expressed by Purkinje cells^23^, which express no or few NMDARs receptors^63, 64^. The mechanism of release involves intracellular acidification of Bergman glia resulting from the proton-permeability of ChR2^53^. In the hippocampus we showed that reducing the pH buffering capacity, which increases glutamate release in the cerebellum^53^, leads to a decrease of ChR2-induced release (Supplementary Fig. 5). This discrepancy could result from the use of different ChR2 mutants (C128S versus H134R) but also from a different pH sensitivity of hippocampal astrocytes and cerebellar Bergman glia, as it has been shown between astrocytes of the brainstem and of the cortex^65^. In the visual cortex, ChR2 activation in astrocytes leads to a Ca^2+^-dependent release of glutamate that increases excitatory and inhibitory transmission through the activation of presynaptic mGluRs^26^. This increase in miniature synaptic currents observed in the neocortex was not affected by an antagonist of purinergic receptors, PPADS^26^, indicating that the autocrine ATP signaling does not control the release of glutamate in the neocortex. Further experiments should be done to clarify whether this difference is the consequence of different properties between hippocampal and cortical astrocytes or to the use of viral versus transgenic approaches.

On the other hand, besides inducing glutamate release, ChR2 stimulation of astrocytes has been previously shown to induce an ATP release. This is the case of brainstem astrocytes in which ChR2 activation leads to a calcium-dependent vesicular release of ATP that propagates through the astrocyte network via P2Y1R activation and eventually depolarizes respiratory neurons^21^. In the hippocampus, ChR2 stimulation of CA1 astrocytes was shown to induce a Ca^2+^-dependent release of ATP that had overall inhibitory effects: it mediates heterosynaptic long-term depression^25^ and excites inhibitory basket cells^29^ via the activation of neuronal P2Y receptors; it inhibits pyramidal neurons via its metabolite adenosine and activation of A1 receptors^29^. We did observe the A1 receptor-mediated inhibition of pyramidal cells but this inhibition appeared after the excitation resulting from NMDAR activation (Fig. 1d). Moreover, in our recording conditions, *i*.*e*. in absence of glutamate receptor antagonists, blocking P2Y1Rs decreased excitation of pyramidal cells. This indicates that the P2YR1-mediated potentiation of astrocyte glutamate release prevails over the P2Y1R-mediated excitation of inhibitory basket cells due to ATP astrocyte release^29^. It should be emphasized that the overall excitation of pyramidal cells we report here develops within few seconds of light stimulation, on a comparable time scale than the excitation reported in the cerebellum with another transgenic approach^23^ and faster than with most viral approaches targeting ChR2 expression in astrocytes^21, 25–29^. Whether this discrepancy results only from a higher proportion of astrocytes expressing ChR2 with the transgenic approaches remains to be clarified. Our observation linking the astrocyte release of glutamate to an autocrine purinergic signaling clearly establishes that glutamate and ATP are co-released by hippocampal astrocytes under the same experimental conditions. This may not be the case in all brain areas but may explain why under specific experimental conditions glutamate or ATP is identified as the predominant gliotransmitter released upon ChR2 stimulation. This will depend on the specific glutamatergic and purinergic neuronal receptors expressed near astrocyte release sites and controlling the neuronal and synaptic activities under investigation.

Potentiation of astrocyte glutamate release by P2Y1R activation has been previously observed in cultures and in acute hippocampal slices^17, 18, 66^. However, the cells releasing ATP in these previous studies were either microglia or neurons. Our results show that in conditions where potential network effects are greatly reduced (TTX, GBZ), selective ChR2 activation in astrocytes triggers astrocyte Ca^2+^ response and glutamate release that are greatly reduced by P2Y1R antagonists and by apyrase. Interestingly, a similar purinergic loop has been shown to contribute to the Ca^2+^ signals induced ChR2 activation in cultured astrocytes^67^. Moreover, we have shown here that the Ca^2+^ response in astrocytes is not affected by the presence of NMDAR antagonists, therefore excluding the possibility that ATP originates from neurons and would be released as a consequence of NMDAR activation^68, 69^, following an initial astrocyte glutamate release. This strongly suggests that ChR2 stimulation in astrocytes triggers a cell autonomous ATP signaling that regulates glutamate release, and it is worth noting that both vesicular and non-vesicular mechanisms of ATP release have been described in astrocytes (reviewed in ref. 70). The positive loop described here could also amplify signals triggered by neuronal and microglial ATP in astrocytes^18, 66^. Yet, autocrine/paracrine ATP signaling through P2Y1R activation supports intercellular signal (including Ca^2+^) propagation through astrocyte syncytia^21, 71, 72^. Although we have not tested for the actual occurrence of a paracrine signaling in our conditions, our results nevertheless suggest that ATP signaling across astrocytes will be accompanied by an enhanced release of glutamate that will increase ambient glutamate and thus tonic activation of neuronal NMDARs. The physiological relevance of this signaling remains to be established and targeting specifically P2Y1R signaling in astrocytes will probably be the best way to address this question. Yet, it is very likely that this ATP-controlled glutamate release from astrocytes will be regulated in pathological conditions. ATP concentration is very often up-regulated in pathological conditions^73, 74^ and the contribution of P2 receptors in the context of neuron-glia interactions has been studied in many pathologies, in particular in pain^75^ and in epilepsy^57^. In the latter case, it is tempting to hypothesize that modification of ATP-P2Y1R autocrine signaling controlling astrocyte glutamate release will contribute to the synchronization of neurons^41, 76^ or to the progressive development of recurrent seizures^77^. Interestingly, large somatic Ca^2+^ transient reminiscent of those triggered here with ChR2 stimulation were observed in cortical astrocytes *in vivo* after pilocarpine-induced status epilepticus^78^. Although these Ca^2+^ signals were attributed to the activation of astrocyte mGluRs, the contribution of P2Y1Rs has not been tested in this study. It would therefore be worth testing the involvement of the purinergic autocrine signaling in these conditions, as it would be worth testing whether this mechanism also controls other forms of glutamate gliotransmission, such as that leading to the generation of slow inward NMDAR-mediated currents^41, 76, 79^.

In conclusion, we have shown here that astrocyte glutamate release is tightly controlled by an autocrine purinergic signaling when astrocytes are selectively activated by optogenetics. This signaling relies on P2Y1R-dependent mobilization of intracellular Ca^2+^ stores that produces Ca^2+^ signals resembling those observed in astrocyte somata in specific physiological and pathological conditions.

## Methods

### Mice

All experiments followed guidelines of the European Union for the care and use of laboratory animals (Council directive 86/609EC) and were approved by the ethics committee of the University of Paris Descartes (registered numbers CEEA34.EA.027.11 and CEEA16-032). C57BL/6 wild-type and transgenic male and female mice were used for the experiments. *Cx30-CreERT2* ^+/*−*^^31^ mice were crossed with hetero- or homozygous Ai32 mice (*B6*;*129S-Gt*(*ROSA*)*26Sor* ^*tm32*(CAG-COP4*H134R/*EYFP*)*Hze*^/*J*, Jackson Labs). These Ai32 mice carry the *ChR2*(*H134R*)*–EYFP* gene in their Gt(ROSA)26Sor locus^80^. CreERT2–mediated induction of ChR2 expression was induced by a single intraperitoneal injection of 1 mg 4-hydroxytamoxifen per approximately 8 g mice weight (Santa Cruz, sc-3542A) around postnatal day 21 (P21). Mice were analyzed at least two weeks after tamoxifen injections.

### Electrophysiology in acute slices

Coronal hippocampal slices were prepared from young adult mice (36–56 day old). Voltage-clamp and current-clamp recordings were obtained from CA1 pyramidal neurons and hippocampal astrocytes. Details on slice preparation, recording conditions and data analysis are provided in Supplementary Information.

### Imaging in acute slices

Astrocytes were loaded with Calcium indicator Rhod-2 AM (9 µM) at room temperature (24 °C) for 1 h with 0.02% Pluronic F-127 (In vitrogen) and 0.6% DMSO (Sigma) in aCSF. Slices were then incubated at least 30 mins in aCSF to wash out the excess of dye before transferring to the imaging chamber. During experiments, slices were perfused with aCSF containing TTX (0.5 µM) and GBZ (10 µM). Fluorescence images were acquired with a custom-built two-photon laser scanning microscope. Details on imaging acquisition and analysis of Ca^2+^ signals are provided in Supplementary Information.

### Optogenetic stimulation

To drive the ChR2 activity in astrocytes we used 470 nm blue light (0.9–6.5 mW/mm^2^) delivered by a LED (Cairn Research, OptoLED) through the 40× water-immersion objective (Olympus). Full field light stimulations of usually 5–20 s were delivered with a 5 min time interval. For photoactivation of astrocytes during calcium imaging, 500 ms pulses of 470 nm light were delivered at 1 Hz during 10–20 s through the light path of the two-photon microscope. An image was acquired in between each pulse of the light stimulation, starting 25 ms after the end of the stimulation, to avoid saturation of the photomultiplier.

In control experiments performed on wild type mice, we verified that light stimulations of similar intensity and duration that the ones used to activate ChR2 in *Cx30-CreERT2:floxed-ChR2-YFP* mice did not induce changes in the holding current recorded in CA1 pyramidal neurons (Supplementary Fig. 1e).

### Immunohistochemistry

The expression of the ChR2-EYFP transgene in *Cx30-CreERT2*:*ChR2-EYFP* was examined by immunolabeling against EYFP and against specific markers of astrocytes (GS, GFAP) and neurons (NeuN). Details are provided in Supplementary Information.

### Statistics

Paired and interleaved experiments were performed; no sample size calculation, no randomization or blinding were performed. No mice were excluded from analysis. Data were analyzed and plotted using SigmaPlot and GraphPad InStat. Comparison of two groups of data was carried out with two-tailed, paired or unpaired, t-test when samples had normal distributions or Wilcoxon matched-pairs test for samples with non-gaussian distributions. To compare means of more than two groups of data, we used One-way repeated measures ANOVA (RM ANOVA) followed by Bonferroni post-hoc test (for Gaussian distribution of data). *P* < 0.05 was considered statistically significant. Statistical data are showed as mean ± SEM or box plots (shoulders of the boxes indicate 25–75% intervals, whiskers indicate the 10th and 90th percentiles, mean-“+” mark or median-thick line of the data are presented within the box). Numbers of cells are given in the parentheses. All graphs are made with CorelDRAW.

### Data availability statement

The data that support the findings of this study are available from the corresponding author upon reasonable request.

## Electronic supplementary material

Supplementary information

## References

[1] Parpura V (2012). Glial cells in (patho)physiology. Journal of Neurochemistry.

[2] Ventura R, Harris KM (1999). Three-dimensional relationships between hippocampal synapses and astrocytes. J. Neurosci..

[3] Witcher MR, Kirov SA, Harris KM (2007). Plasticity of perisynaptic astroglia during synaptogenesis in the mature rat hippocampus. Glia.

[4] Schikorski T, Stevens CF (1997). Quantitative ultrastructural analysis of hippocampal excitatory synapses. J. Neurosci..

[5] Grosche J (1999). Microdomains for neuron-glia interaction: parallel fiber signaling to Bergmann glial cells. Nat.Neurosci..

[6] Marcaggi P, Attwell D (2004). Role of glial amino acid transporters in synaptic transmission and brain energetics. Glia.

[7] Schipke CG, Kettenmann H (2004). Astrocyte responses to neuronal activity. Glia.

[8] Panatier A, Robitaille R (2016). Astrocytic mGluR5 and the tripartite synapse. Neuroscience.

[9] Araque A (2014). Gliotransmitters travel in time and space. Neuron.

[10] Gundersen V, Storm-Mathisen J, Bergersen LH (2015). Neuroglial Transmission. Physiol. Rev..

[11] Velez-Fort M, Audinat E, Angulo MC (2012). Central Role of GABA in Neuron-Glia Interactions. Neurosci..

[12] Hamilton NB, Attwell D (2010). Do astrocytes really exocytose neurotransmitters?. Nat.Rev.Neurosci..

[13] Agulhon C (2008). What is the role of astrocyte calcium in neurophysiology?. Neuron.

[14] Zorec, R. *et al*. Astroglial excitability and gliotransmission: an appraisal of Ca^2+^ as a signalling route. *ASN*. *Neuro*. **4** (2012). 10.1042/AN20110061 PMC3310306 22313347

[15] Bazargani N, Attwell D (2016). Astrocyte calcium signaling: the third wave. Nat. Neurosci..

[16] Sherwood MW (2017). Astrocytic IP _3_ Rs: Contribution to Ca^2+^ signalling and hippocampal LTP. Glia.

[17] Domercq M (2006). P2Y1 receptor-evoked glutamate exocytosis from astrocytes: control by tumor necrosis factor-alpha and prostaglandins. J.Biol.Chem..

[18] Jourdain P (2007). Glutamate exocytosis from astrocytes controls synaptic strength. Nat.Neurosci..

[19] Hines DJ, Haydon PG (2014). Astrocytic adenosine: from synapses to psychiatric disorders. Philos. Trans. R. Soc. B Biol. Sci..

[20] Li D, Agulhon C, Schmidt E, Oheim M, Ropert N (2013). New tools for investigating astrocyte-to-neuron communication. Front Cell Neurosci..

[21] Gourine AV (2010). Astrocytes control breathing through pH-dependent release of ATP. Science (80-.)..

[22] Gradinaru V, Mogri M, Thompson KR, Henderson JM, Deisseroth K (2009). Optical deconstruction of parkinsonian neural circuitry. Science (80-.)..

[23] Sasaki T (2012). Application of an optogenetic byway for perturbing neuronal activity via glial photostimulation. Proc.Natl.Acad.Sci.U.S.A.

[24] Li D, Herault K, Isacoff EY, Oheim M, Ropert N (2012). Optogenetic activation of LiGluR-expressing astrocytes evokes anion channel-mediated glutamate release. J Physiol.

[25] Chen J (2013). Heterosynaptic long-term depression mediated by ATP released from astrocytes. Glia.

[26] Perea G, Yang A, Boyden ES, Sur M (2014). Optogenetic astrocyte activation modulates response selectivity of visual cortex neurons *in vivo*. Nat. Commun..

[27] Berlinguer-Palmini R (2014). Arrays of microLEDs and astrocytes: biological amplifiers to optogenetically modulate neuronal networks reducing light requirement. PLoS One.

[28] Poskanzer KE, Yuste R (2016). Astrocytes regulate cortical state switching *in vivo*. Proc. Natl. Acad. Sci. USA.

[29] Tan Z (2017). Glia-derived ATP inversely regulates excitability of pyramidal and CCK-positive neurons. Nat. Commun..

[30] Fellin T, Pascual O, Haydon PG (2006). Astrocytes coordinate synaptic networks: balanced excitation and inhibition. Physiol..

[31] Slezak M (2007). Transgenic mice for conditional gene manipulation in astroglial cells. Glia.

[32] Lalo U (2014). Exocytosis of ATP from astrocytes modulates phasic and tonic inhibition in the neocortex. PLoS Biol..

[33] Cunha RA, Ribeiro JA (2000). ATP as a presynaptic modulator. Life Sci..

[34] Burnstock G (2007). Purine and pyrimidine receptors. Cell. Mol. Life Sci..

[35] Lalo U, Palygin O, Verkhratsky A, Grant SGN, Pankratov Y (2016). ATP from synaptic terminals and astrocytes regulates NMDA receptors and synaptic plasticity through PSD-95 multi-protein complex. Sci. Rep..

[36] Perea G, Araque A (2007). Astrocytes potentiate transmitter release at single hippocampal synapses. Science.

[37] Le Meur K, Galante M, Angulo MC, Audinat E (2007). Tonic activation of NMDA receptors by ambient glutamate of non-synaptic origin in the rat hippocampus. J. Physiol..

[38] Jabaudon D (1999). Inhibition of uptake unmasks rapid extracellular turnover of glutamate of nonvesicular origin. Proc.Natl.Acad.Sci.U.S.A.

[39] Cavelier P, Attwell D (2005). Tonic release of glutamate by a DIDS-sensitive mechanism in rat hippocampal slices. J.Physiol.

[40] Woo DH (2012). TREK-1 and Best1 channels mediate fast and slow glutamate release in astrocytes upon GPCR activation. Cell.

[41] Fellin T (2004). Neuronal synchrony mediated by astrocytic glutamate through activation of extrasynaptic NMDA receptors. Neuron.

[42] Fischer G (1997). Ro 25-6981, a highly potent and selective blocker of N-methyl-D-aspartate receptors containing the NR2B subunit. Characterization *in vitro*. J. Pharmacol. Exp. Ther..

[43] Papouin T (2012). Synaptic and extrasynaptic NMDA receptors are gated by different endogenous coagonists. Cell.

[44] Vergnano AM (2014). Zinc dynamics and action at excitatory synapses. Neuron.

[45] Paoletti P, Bellone C, Zhou Q (2013). NMDA receptor subunit diversity: impact on receptor properties, synaptic plasticity and disease. Nat. Rev. Neurosci..

[46] Izumi Y, Auberson YP, Zorumski CF (2006). Zinc modulates bidirectional hippocampal plasticity by effects on NMDA receptors. J. Neurosci..

[47] Kew JN, Trube G, Kemp JA (1996). A novel mechanism of activity-dependent NMDA receptor antagonism describes the effect of ifenprodil in rat cultured cortical neurones. J.Physiol.

[48] Nagel G (2005). Light activation of Channelrhodopsin-2 in excitable cells of caenorhabditis elegans triggers rapid behavioral responses. Curr. Biol..

[49] Nagel G (2003). Channelrhodopsin-2, a directly light-gated cation-selective membrane channel. Proc. Natl. Acad. Sci. USA.

[50] Caldwell JH (2008). Increases in intracellular calcium triggered by channelrhodopsin-2 potentiate the response of metabotropic glutamate receptor mGluR7. J. Biol. Chem..

[51] Verkhratsky A, Burnstock G (2014). Purinergic and glutamatergic receptors on astroglia. Adv. Neurobiol..

[52] Brown, S. G. *et al*. Activity of Novel Adenine Nucleotide Derivatives as Agonists and Antagonists at Recombinant Rat P2X Receptors. *Drug Dev Res* **491002** (2000). 10.1002/1098-2299(200004)49:4<253::AID-DDR4>3.0.CO;2-1 PMC3393598 22791931

[53] Beppu K (2014). Optogenetic countering of glial acidosis suppresses glial glutamate release and ischemic brain damage. Neuron.

[54] Peoples RW, Li C (1998). Inhibition of NMDA-gated ion channels by the P2 purinoceptor antagonists suramin and reactive blue 2 in mouse hippocampal neurones. Br. J. Pharmacol..

[55] Sah P, Hestrin S, Nicoll RA (1989). Tonic activation of NMDA receptors by ambient glutamate enhances excitability of neurons. Science (80-.)..

[56] Lin JY, Lin MZ, Steinbach P, Tsien RY (2009). Characterization of engineered channelrhodopsin variants with improved properties and kinetics. Biophys. J..

[57] Rassendren, F. & Audinat, E. Purinergic signaling in epilepsy. *J*. *Neurosci*. *Res*. **94** (2016). 10.1002/jnr.23770 27302739

[58] Darby M, Kuzmiski JB, Panenka W, Feighan D, Macvicar BA (2003). ATP released from astrocytes during swelling activates chloride channels. J. Neurophysiol..

[59] Ding F (2013). alpha1-Adrenergic receptors mediate coordinated Ca^2+^ signaling of cortical astrocytes in awake, behaving mice. Cell Calcium.

[60] Paukert M (2014). Norepinephrine controls astroglial responsiveness to local circuit activity. Neuron.

[61] Rusakov DA, Bard L, Stewart MG, Henneberger C (2014). Diversity of astroglial functions alludes to subcellular specialisation. Trends in Neurosciences.

[62] Volterra A, Liaudet N, Savtchouk I (2014). Astrocyte Ca^2+^ signalling: an unexpected complexity. Nat. Rev. Neurosci..

[63] Crepel, F. & Audinat, E. Excitatory amino acids receptors of cerebellar purkinje cells: Development and plasticity. *Prog*. *Biophys*. *Mol*. *Biol*. **55** (1991). 10.1016/0079-6107(91)90010-p 1647540

[64] Piochon C (2007). NMDA receptor contribution to the climbing fiber response in the adult mouse Purkinje cell. J Neurosci.

[65] Kasymov V (2013). Differential sensitivity of brainstem versus cortical astrocytes to changes in pH reveals functional regional specialization of astroglia. J. Neurosci..

[66] Pascual O, Ben Achour S, Rostaing P, Triller A, Bessis A (2012). Microglia activation triggers astrocyte-mediated modulation of excitatory neurotransmission. Proc. Natl. Acad. Sci..

[67] Figueiredo M (2014). Comparative analysis of optogenetic actuators in cultured astrocytes. Cell Calcium.

[68] Eyo UB (2014). Neuronal Hyperactivity Recruits Microglial Processes via Neuronal NMDA Receptors and Microglial P2Y12 Receptors after Status Epilepticus. J. Neurosci..

[69] Dissing-Olesen L (2014). Activation of neuronal NMDA receptors triggers transient ATP-mediated microglial process outgrowth. J. Neurosci..

[70] Rassendren F, Audinat E (2016). Purinergic signaling in epilepsy. J. Neurosci. Res..

[71] Haas B (2006). Activity-dependent ATP-waves in the mouse neocortex are independent from astrocytic calcium waves. Cereb.Cortex.

[72] Delekate A (2014). Metabotropic P2Y1 receptor signalling mediates astrocytic hyperactivity *in vivo* in an Alzheimer’s disease mouse model. Nat. Commun..

[73] Rodrigues RJ, Tome AR, Cunha RA (2015). ATP as a multi-target danger signal in the brain. Front Neurosci..

[74] Franke H, Verkhratsky A, Burnstock G, Illes P (2012). Pathophysiology of astroglial purinergic signalling. Purinergic Signalling.

[75] Tsuda M, Beggs S, Salter MW, Inoue K (2013). Microglia and intractable chronic pain. Glia.

[76] Angulo MC, Kozlov AS, Charpak S, Audinat E (2004). Glutamate released from glial cells synchronizes neuronal activity in the hippocampus. J. Neurosci..

[77] Clasadonte J, Dong J, Hines DJ, Haydon PG (2013). Astrocyte control of synaptic NMDA receptors contributes to the progressive development of temporal lobe epilepsy. Proc. Natl. Acad. Sci. USA.

[78] Ding S (2007). Enhanced astrocytic Ca^2+^ signals contribute to neuronal excitotoxicity after status epilepticus. J Neurosci..

[79] Parri HR, Gould TM, Crunelli V (2001). Spontaneous astrocytic Ca^2+^ oscillations *in situ* drive NMDAR-mediated neuronal excitation. Nat.Neurosci..

[80] Madisen L (2012). A toolbox of Cre-dependent optogenetic transgenic mice for light-induced activation and silencing. Nat. Neurosci..

