# Supplementary material for: An autocrine purinergic signaling controls astrocyte-induced neuronal excitation
Source: Sci Rep. 2017 Sep 12;7:11280. doi: 10.1038/s41598-017-11793-x (PMC5595839; doi:10.1038/s41598-017-11793-x)
Supplement: Supplementary file 1 — Supplementary information [file 41598_2017_11793_MOESM1_ESM.pdf]

Supplementary information

An autocrine purinergic signaling controls astrocyte-induced neuronal excitation

Weida Shen[1ǂ], Ljiljana Nikolic[1ǂ], Claire Meunier[1], Frank Pfrieger[2], Etienne Audinat[1*]

[1]Inserm U1128, Paris Descartes University, 75006 Paris France

[2]Institute of Cellular and Integrative Neurosciences, CNRS UPR 3212, University of Strasbourg, 67084 Strasbourg, France.

[ǂ] These authors contributed equally to this work

[*]Corresponding author: Etienne Audinat

Inserm U1128, Université Paris Descartes

45 rue des Saints Pères, 75006 Paris, France

E-mail: etienne.audinat@parisdescartes.fr

Supplementary Materials and Methods

Electrophysiological recordings

36-to-56 day-old mice were anaesthetized with isoflurane, humanely killed by cervical dislocation and decapitated. Coronal hippocampal slices (300 µm) were cut in an oxygenated (5% CO_2 and 95% O_2) ice-cold protective extracellular solution containing (in mM): 93 NMDG, 2.5 KCl, 1.2 NaH_2PO_4, 30 $NaHCO_3$, 20 HEPES, 2 thiourea, 25 D-glucose, 5 sodium ascorbate, 3 sodium pyruvate, 10 $MgCl_2$, and 0.5 $CaCl_2$ (pH 7.3, 310 mOsm). After cutting, slices were first incubated in the same protective extracellular solution for 7 min at 34°C and then incubated at 34°C for 30 min in regular artificial cerebro-spinal fluid (aCSF; pH 7.4, 310 mOsm) containing (in mM): 126 NaCl, 2.5 KCl, 26 $NaHCO_3$, 1.25 NaH_2PO_4, 20 mM D-glucose, 1 sodium pyruvate, 1 $MgCl_2$, and 2 $CaCl_2$. The slices were then maintained at room temperature (RT, 22-24°C) for 0.5–5h in the regular oxygenated aCSF until the start of recordings.

Individual slices were transferred to a recording chamber perfused with regular aCSF at 3 ml/min. Whole-cell patch-clamp recordings were performed from CA1 pyramidal neurons in current- and voltage-clamp mode. For current-clamp recordings pipettes (3-5 MΩ) were filled with a solution containing in mM: 125 K-Gluconate, 2 $MgCl_2$, 10 HEPES, 0.4 Na^+-GTP, 4 ATP-Na_2, 10 Phosphocreatine disodium salt, 10 KCl, 0.5 EGTA (pH 7.3-7.4, 280-290 mOsm). For voltage-clamp recordings pipettes contained (in mM): 125 $CsMeSO_3$, 10 HEPES, 10 EGTA, 8 TEA-Cl, 5 4-AP, 0.4 GTP-Na, 4 ATP-Na_2, 1 $CaCl_2$ and 1 $MgCl_2$ (pH 7.3-7.4, 280-290 mOsm). To perform whole-cell patch-clamp recordings from hippocampal astrocytes we used pipettes (7-8 MΩ) filled with a solution containing (in mM): 130 K-Gluconate, 20 HEPES, 3 ATP-Na_2, 10 D-Glucose, 1 $MgCl_2$, 0.2 EGTA (pH 7.3-7.4, 280-290 mOsm). In a subset of experiments 40 mM BAPTA was included and substituted for K-Gluconate. Patch-clamp recordings were performed using either Axopatch 200B or 700B amplifiers (Molecular Devices). Data were filtered at 5 kHz or 6 kHz and sampled at 10 kHz, then analyzed off-line using pClamp 10.4 software (Molecular Devices). Series resistances were monitored using -10 mV pules. For all recordings performed with K-gluconate or Cs-gluconate in the pipette, potentials were corrected for a junction potential of -10 mV. Data were accepted if the series resistances changed by < 20% during the whole experiment. Experiments were performed at room temperature (22-24°C, RT), except for the recordings of mEPSCs and for current-clamp recordings that were performed at 33°C.

To record tonic NMDA current neurons were clamped at +40 mV, whereas mEPSCs were recorded at -70 mV in aCSF including 0.5-1 µM TTX and 10 µM GBZ. All pharmacological experiments on mEPSCs were compared with interleaved control experiments.

In experiments with exogenous zinc applications designed to block NMDARs, tricine (10 mM) was used to buffer zinc, and free zinc concentration was calculated to be 300 nM. For recording the NMDAR-mediated component of EPSCs at Schaffer collateral (SC)-CA1 synapses, slices were continually perfused with NBQX (10 µM) and Gabazine (10 µM) and the recorded cells were held at +40 mV. SC fibers were stimulated at 0.05 or 0.016 Hz using a theta glass capillary (TGC150-10; Harvard Apparatus) filled with regular aCSF.

For experiments designed to inhibit intracellular Ca^{2+} stores, slices were incubated for 30-60 min with 1 µM thapsigargin at 32°C. For experiments aiming at inhibiting the vacuolar H^{+}-ATPase, the slices were incubated for 3h at 34°C in the aCSF containing 4 µM of the inhibitor bafilomycin A1. For each experiment using thapsigargin or bafilomycin A1, interleaved control slices from the same mouse were incubated in the same conditions but without thapsigargin or bafilomycin A1. In all bafilomycin A1-treated slices we verified that no synaptic currents occurred spontaneously or when adding 10 mM potassium chloride to the aCSF.

In experiments designed to manipulate the capacity of cells to buffer pH, 26 mM, $NaHCO_3$ was replaced by 5 mM HEPES and 26 mM NaCl (pH was adjusted to the same level of normal aCSF, pH 7.4, 310 mOsm). This solution was bubbled with pure oxygen.

Off-line analysis was performed using pClamp10.4 software (Molecular Devices). Action potentials were counted (number of spikes) either during 5 s or 10 s just before and during the light stimulation (for excitation) or during 10 s periods just before and 2.5 s after the end of the light stimulation (for inhibition). Miniature EPSCs (mEPSCs) were counted by setting the event detection threshold at twice the standard deviation of the noise, sorted using 2 s (bins) and counted (frequency) and measured (amplitude) during 18-20 s periods just before (bef.), during (on) and 30 sec after the light stimulation (off). For each cell, the mean mEPSC frequency and amplitude were calculated for each of these 3 time periods (before, during and after). The amplitude of the tonic NMDA current was determined as the difference between the mean of the holding current within 100 ms just before photostimulation and the mean of the evoked current within 100 ms at the end of the blue light stimulation.

Calcium imaging

Fluorescence was imaged using a 40×water-immersion objective (Olympus) with a custom-built two-photon laser scanning microscope. EYFP and the Rhod-2 fluorophore were excited at 850 nm, the two fluorescence signals were separated by a dichroic (560 nm) and the EYFP signal was further filtered through a 525±7 nm bandpass filter (Semrock). Images were acquired at 1 Hz in frame mode (500 ms per frame) with a custom-made software (LabVIEW, National Instruments). Image stacks (30-40 optical sections, 1 μM z-spacing) were acquired after every experiment to aid the identification of astrocytes on the basis of EYFP expression.

Ca^{2+} signals were analyzed in ROIs covering the entire soma of astrocytes. Normalized changes in Rhod-2 fluorescence were calculated as $\Delta F/F = (F - F0) / F0$. The threshold for measuring the onset of light-evoked Ca^{2+} transients was set where the change in F relative to F0 was greater than 2 × s.d. of the baseline signal for at least 5 s. Ca^{2+} signals induced by light stimulation were then quantified by measuring the integral of ΔF/F and dividing it by the duration of the light stimulation to give a mean ΔF/F during the response period. Peak amplitude of Ca^{2+} signals is represented by the first signal peak overshooting the detection threshold of 5 s.d. A minimum of two stable responses were acquired before testing the effects of any drug.

Immunohistochemistry

Cx30-CreERT2:ChR2-EYFP mice (P36-56) were anesthetized with sodium pentobarbital (50 mg/kg) and then perfused transcardially with PBS followed by 4% paraformaldehyde (PFA) in 0.15 M phosphate buffer. Brains were removed and fixed in 4% PFA overnight. Brains were cut in 50 μm sections using a vibrating microtome. After several washes in phosphate-buffered saline (PBS), sections were placed for 1h in a blocking solution containing 4% normal goat serum (NGS, Sigma Aldrich) and 1% Triton X-100 (Sigma Aldrich) at room temperature. The following primary antibodies were used: chicken anti-EYFP (1:500 or 1:1000, Invitrogen A10262) mouse anti-glutamine synthetase (1:500, Miilipore MAB302), guinea pig anti-NeuN (Milipore ABN90), rabbit anti-GFAP (1:1000, ABCAM AB7260). Incubation with primary antibodies overnight at 4°C was followed by several washes and an incubation with fluorescently labeled secondary antibodies: goat anti-chicken Alexa 488 (1:250, Invitrogen, A-11039), goat anti-mouse Alexa 555 (1:250, Invitrogen, A11030), goat anti-guinea pig Alexa 633 (1:250, Invitrogen A21105), goat anti-rabbit DyLight 550 (1:500, Grisera AS111781) for 2 or 2.5 h in dark at room temperature. Sections were rinsed and mounted in Vectashield (Vector Laboratories) for confocal microscopy (Zeiss LSM-510 or LSM-710). Images were analyzed in ImageJ (NIH). Colocalisation was determined by using

Colocalisation Threshold method in ImageJ. Astrocyte cell bodies were counted manually using Cell Counter ImageJ plugin.

Drugs and chemicals

50 µM D-(-)-2-Amino-5-phosphonopentanoic acid (D-AP 5), 10 µM 2,3-Dioxo-6-nitro-1,2,3,4-tetrahydrobenzo[f]quinoxaline-7-sulfonamide (NBQX), 10 µM SR95531 (Gabazine) were purchased from HelloBio. 0.5-1 µM Tetrodotoxin citrate (TTX), 4 µM Bafilomycin A1, 10 µM disodium salt, 2'-Deoxy-N6-methyladenosine 3',5'-bisphosphate tetrasodium salt (MRS 2179), 1 µM Thapsigargin, 9 µM Rhod-2 AM, were purchased from Abcam. 100 µM DL-*threo*-β-Benzyloxyasparticacid (DL-TBOA), 50 µM 2-Methyl-6-(phenylethynyl)pyridine hydrochloride (MPEP), 100 µM Pyridoxalphosphate-6-azophenyl-2',4'-disulfonic acid tetrasodium salt (PPADS), 100 µM (RS)-α-Cyclopropyl-4-phosphonophenylglycine (CPPG), 100 µM (S)-(+)-α-Amino-4-carboxy-2-methylbenzeneacetic acid (LY 367385), 50 µM Cyclothiazide (CTZ), 300 nM (αR,βS)-α-(4-Hydroxyphenyl)-β-methyl-4-(phenylmethyl)-1-piperidinepropanol maleate (Ro 25-6981), 50 µM 7-Chlorokynurenic acid (7Cl-KYN), 100 µM glycine, 10 µM D-serine, 2-[2-[4-(4-Nitrobenzyloxy)phenyl]ethyl]isothiourea mesylate (KB-R7943 mesylate), 300 nM 8-Cyclopentyl-1,3-dipropylxanthine (DPCPX), Pyridoxalphosphate-6-azophenyl-2',4'-disulfonic acid tetrasodium salt (PPADS), N-Methyl-D-aspartic acid (NMDA), 100 µM 5-nitro-2-(3-phenylpropyl amino) benzoic acid (NPPB), 20 µM N-[1-[[(Cyanoamino)(5-quinolinylamino)methylene]amino]-2,2-dimethylpropyl]-3,4-dimethoxybenzeneacetamide (A740003) were purchased from Tocris-Cookson. 25 U/mL apyrase was purchased from Sigma. 40 mM 1,2-Bis(2-aminophenoxy)ethane-N,N,N',N'-tetraacetic acid (BAPTA) was purchased from Thermo Fisher Scientific. 4,4',4'',4'''-[Carbonylbis(imino-5,1,3-benzenetriyl-bis(carbonylimino))]tetrakis-1,3-benzenedisulfonic acid, octasodium salt (NF 449) was purchased from Cayman Chemicals.

Supplementary Figures

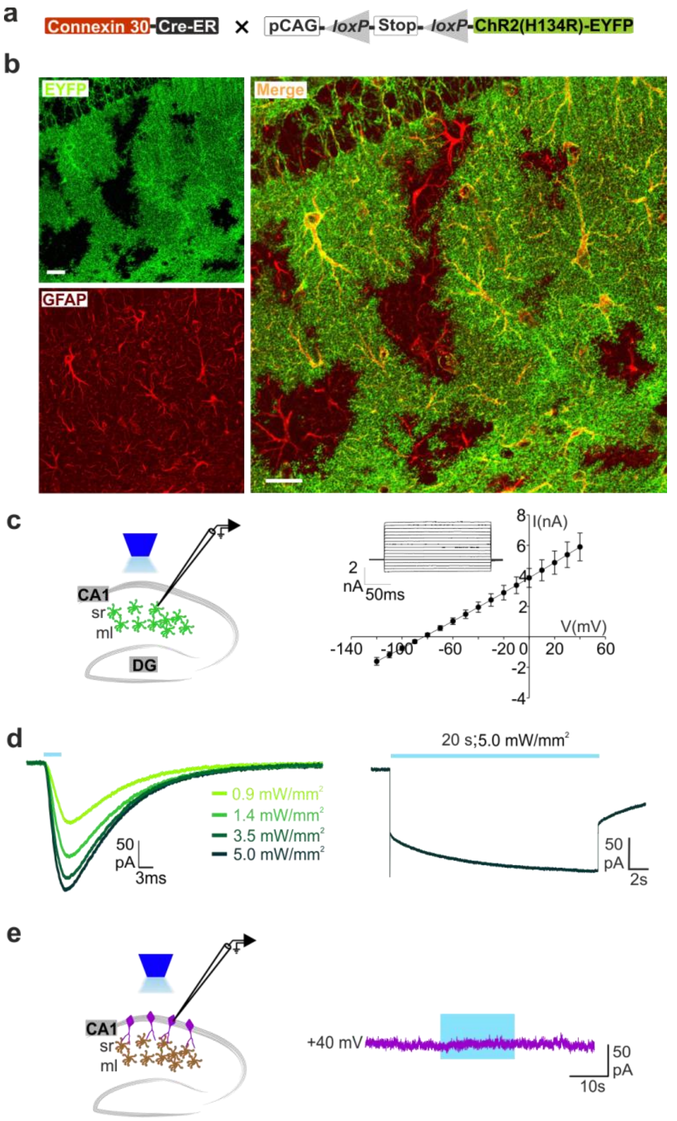

Supplementary Figure 1: ChR2 is specifically expressed in astrocytes in Cx30-Cre:ChR2-EYFP mouse. (**a**) Schematic representation of the genetic strategy for the expression of ChR2 in astrocytes. Mice expressing tamoxifen-inducible Cre recombinase (Cre-ER) under the Connexin 30 promoter were crossed with Ai32 mice expressing ChR2 and EYFP (H134R-EYFP) under the CAG promoter (pCAG) with a stop sequence flanked by loxP sites. (**b**) Confocal images (z stack 3.5 µm, 8 optical slices, 3 animals) of immunolabelings showing the expression of ChR2-EYFP (green), and its co-localization with astrocyte specific marker glial fibrillary acidic protein (GFAP, red) in the stratum radiatum of CA1. Scale bar 20 µm. (**c**) Left, schematic representation of the experimental setting for whole-cell recordings of ChR2$^+$ astrocytes (green) during light stimulation (light blue). Right, ChR2$^+$ astrocytes have a typical linear current/voltage relationship. The inset show the current traces in response to the voltage steps depicted on the graph of the I/V curve (16 cells, 10 animals). (**d**) Light pulses reliably induce inward currents in ChR2$^+$ astrocytes. Left, increasing membrane currents recorded from ChR2$^+$ astrocytes in response to brief photostimulations of increasing intensity. Right, membrane current recorded in a ChR2$^+$ astrocyte in response to a 20s light stimulation. (**e**) Left, scheme for whole-cell recordings of CA1 neurons in wild-type mouse. Right, representative trace showing that blue light has no effect on the holding current in wild-type mouse.

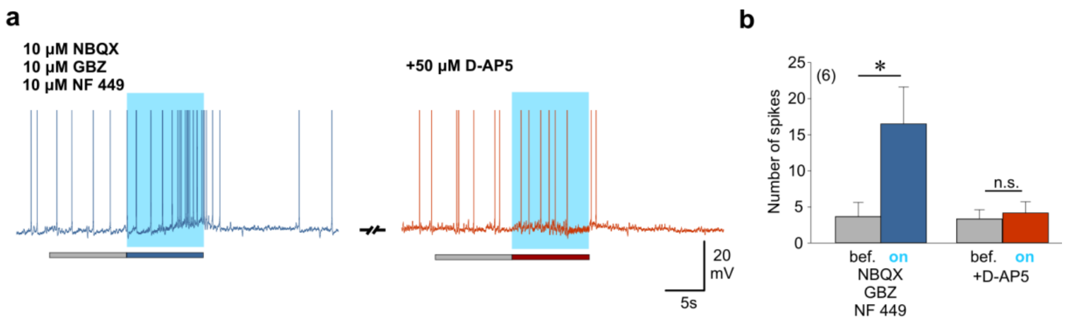

Supplementary Figure 2: $GABA_A$ and P2X receptors are not involved in the increase in action potential frequency evoked by astrocyte photostimulation. (a) Current clamp traces illustrating the responses of a CA1 pyramidal neuron to light activation of astrocytes in the presence of NBQX (10 µM), gabazine (GBZ, 10 µM), NF 449 (10 µM) and after application of 50 µM D-AP5. A constant depolarizing current was injected throughout the recording to maintain the membrane potential near the action potential threshold. **(b)** Number of action potentials within 10 s time windows before and during photostimulation of astrocytes in the presence of NBQX, GBZ and NF 449 without (left) and with (right) D-AP5 (NBQX, GBZ, NF 449: $t(5)=3.592$, $P=0.016$; + D-AP5: $t(5)=1.746$, $P=0.141$; 6 cells, 3 animals, paired t-test).

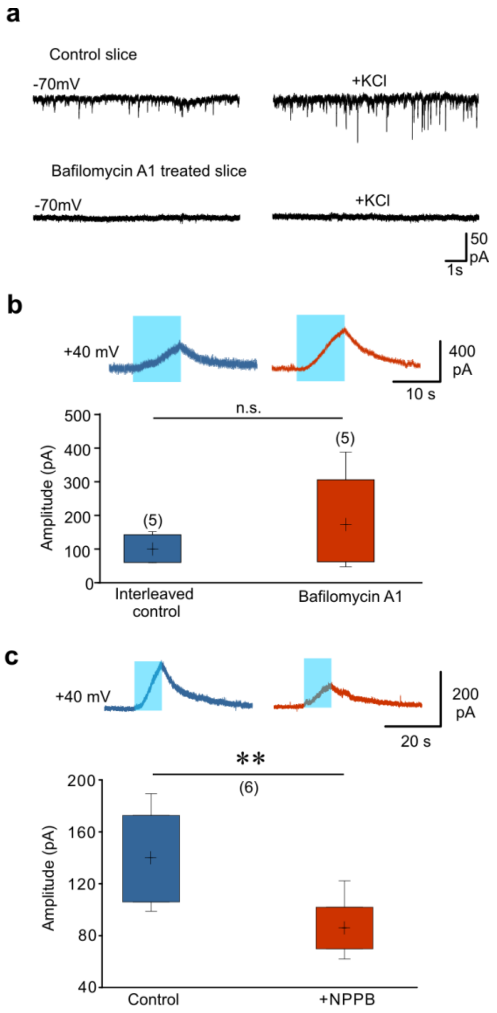

Figure 3. Mechanism of glutamate release by light-activated astrocytes. (a) Comparison of the synaptic activity and the effect of bath applied 10 mM KCl in CA1 pyramidal neurons (Vh=-70mV) in control (upper traces) and bafilomycin A1 (4 µM) treated slices (lower traces). **(b)** Light-evoked tonic NMDAR-mediated currents in control and bafilomycin A1 treated slices (Vh=+40 mV). The amplitude of light-evoked tonic NMDAR-mediated currents did not differ between pyramidal cells of bafilomycin A1 treated slices and of interleaved controls (t(8)=1.132, P=0.290, 10 cells, 3 animals, unpaired t-test). **(c)** Blocking of Ca^{2+}-activated anion channels with NPPB (100 µM) reduced light-evoked tonic NMDAR-mediated currents (t(5)=6.48, P=0.0013; 6 cells, 3 animals, paired t-test).

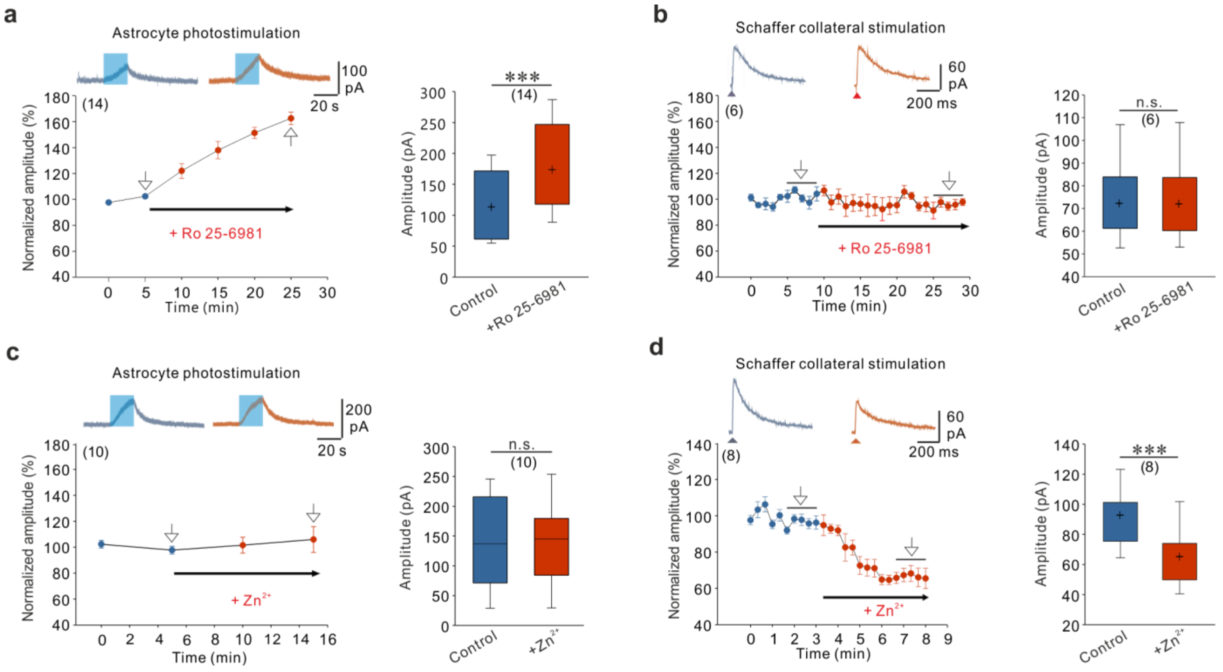

Supplementary Figure 4: Glutamate released by astrocyte photostimulation targets GluN2B-containing NMDARs on CA1 pyramidal neurons. (**a, b**) Time course (left) and mean changes (right) induced by the modulator of GluN2B-containing NMDARs, Ro 25-6981 (300 nM), on the amplitude of tonic NMDAR-mediated current evoked by astrocyte photostimulation (**a**: t(13)=10.801, P<0.0001, 14 cells, 5 animals) and on the amplitude of the NMDAR-mediated component of EPSCs evoked by Schafer collateral stimulation in CA1 neurons (**b:** t(5)=0.310, P=0.769, 6 cells, 3 animals), paired t-test. (**c, d**) Time course (left) and mean changes (right) induced by the modulator of GluN2A-containing NMDARs, Zn^{2+} (300 nM) on the amplitude of tonic NMDAR-mediated currents evoked by astrocyte photostimulation (**c**: z=0.663, P=0.557, 10 cells, 5 animals, Wilcoxon test) and on the amplitude of the NMDAR-mediated component of EPSCs evoked by Schafer collateral stimulation in CA1 neurons (**d**: t(7)=11.207, P<0.0001, 8 cells, 2 animals, paired t-test). Representative traces are shown above the graphs at time points indicated by the arrows.

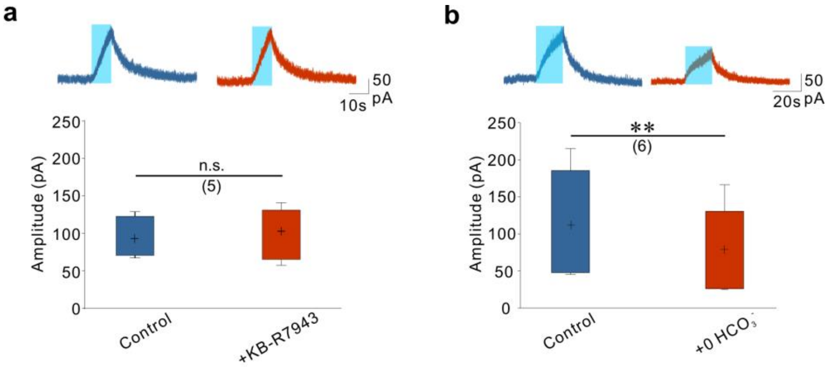

Supplementary Figure 5. Astrocyte glutamate release is not driven by a reverse operation of the Na^+/Ca^{2+} exchanger or by intracellular acidification. (**a**) Blocking the reverse operation mode of the Na^+/Ca^{2+} exchanger with 5 µM KB-R7943 did not change the amplitude of the tonic NMDA current of a CA1 neurons evoked by astrocyte photostimulation ($t(4)=1.056$, $P=0.351$, 5 cells, 4 animals, paired t-test). (**b**) Amplitude of the tonic NMDAR-mediated current triggered by ChR2 stimulation in CA1 pyramidal neurons is reduced after exchanging $NaHCO_3$ (26 mM) for HEPES (5 mM) in aCSF ($t(5)=5.131$, $P=0.004$, 6 cells, 3 animals, paired t-test). This manipulation will lead to a reduction of intracellular $NaHCO_3$ concentration and thus of pH buffer capacity of the cells. If astrocyte glutamate release was driven by the entry of protons through ChR2 channels, *i.e.* acidification, this should increase NMDAR-mediated currents. Representative traces of the NMDAR-mediated tonic currents are shown above the graphs.

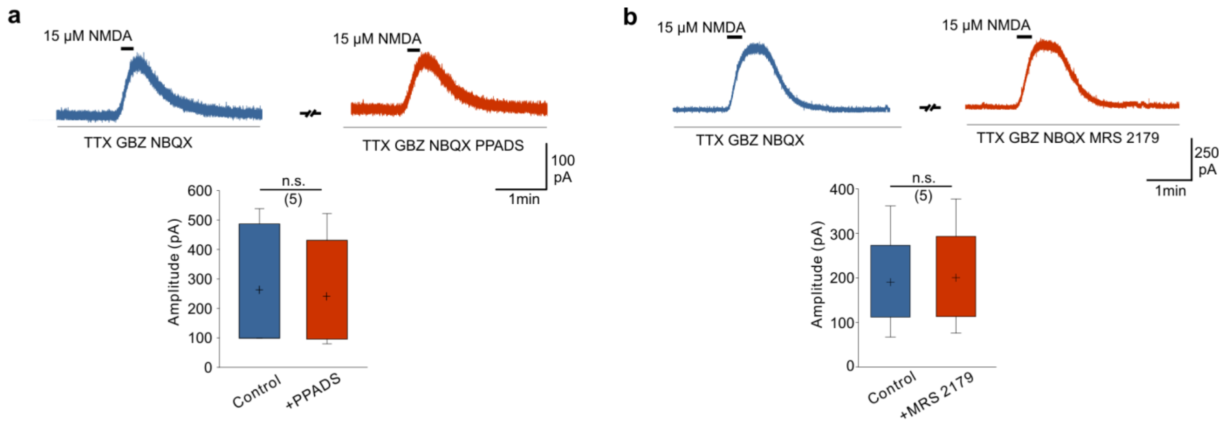

Supplementary Figure 6: P2R antagonists PPADS and MRS 2179 have no direct effect on NMDA receptors. (a) Currents induced by bath applications of NMDA (15 µM) in control conditions and in the presence of PPADS (100 µM; **a**) or MRS2179 (10 µM; **b**) in CA1 pyramidal cells held at +40 mV. The amplitude of tonic NMDA current is not affected by PPADS (t(4)=0.452, P=0.675, 5 cells 2 animals, paired t-test) nor by MRS 2179 (t(4)=1.918, P=0.128, 5 cells from 2 animals, paired t-test).
